# Explaining Bacterial Dispersion on Leaf Surfaces with an Individual-Based Model (PHYLLOSIM)

**DOI:** 10.1371/journal.pone.0075633

**Published:** 2013-10-04

**Authors:** Annemieke van der Wal, Robin Tecon, Jan-Ulrich Kreft, Wolf M. Mooij, Johan H. J. Leveau

**Affiliations:** 1 Department of Microbial Ecology, Netherlands Institute of Ecology (NIOO-KNAW), Wageningen, The Netherlands; 2 Department of Plant Pathology, University of California Davis, Davis, California, United States of America; 3 Centre for Systems Biology, School of Biosciences, The University of Birmingham, Birmingham, United Kingdom; 4 Department of Aquatic Ecology, Netherlands Institute of Ecology (NIOO-KNAW), Wageningen, The Netherlands; 5 Department of Aquatic Ecology and Water Quality Management, Wageningen University, Wageningen, The Netherlands; National Council of Research (CNR), Italy

## Abstract

We developed the individual-based model PHYLLOSIM to explain observed variation in the size of bacterial clusters on plant leaf surfaces (the phyllosphere). Specifically, we tested how different ‘waterscapes’ impacted the diffusion of nutrients from the leaf interior to the surface and the growth of individual bacteria on these nutrients. In the ‘null’ model or more complex ‘patchy’ models, the surface was covered with a continuous water film or with water drops of equal or different volumes, respectively. While these models predicted the growth of individual bacterial immigrants into clusters of variable sizes, they were unable to reproduce experimentally derived, previously published patterns of dispersion which were characterized by a much larger variation in cluster sizes and a disproportionate occurrence of clusters consisting of only one or two bacteria. The fit of model predictions to experimental data was about equally poor (<5%) regardless of whether the water films were continuous or patchy. Only by allowing individual bacteria to detach from developing clusters and re-attach elsewhere to start a new cluster, did PHYLLOSIM come much closer to reproducing experimental observations. The goodness of fit including detachment increased to about 70–80% for all waterscapes. Predictions of this ‘detachment’ model were further supported by the visualization and quantification of bacterial detachment and attachment events at an agarose-water interface. Thus, both model and experiment suggest that detachment of bacterial cells from clusters is an important mechanism underlying bacterial exploration of the phyllosphere.

## Introduction

Plant foliage (also known as the phyllosphere) supports large populations of bacteria on its surface, as high as 10^7^ per square centimeter [1], [2]. Under the microscope, these bacterial colonizers are typically seen organized in aggregates or clusters [3]. In a key experimental study, Monier and Lindow [4] found that up to 50% of *Pseudomonas syringae* bacteria on bean leaves were located in clusters of 10^3^ cells or more after 8 days of incubation. To explain this highly clumped dispersion of bacteria on leaf surfaces, Monier [5] proposed a conceptual model which assumes that 1) immigrant bacteria arrive on the leaf as single cells in a random spatial pattern and 2) only a few sites on the leaf offer conditions that allow bacterial growth. The growth of cells in these conducive sites, but not of those in other sites, results in a transition from an initial pattern of randomly distributed single immigrant cells to a pattern of clumped distribution of bacteria in clusters that represent progeny of successful immigrants [6]. This model of leaf colonization has been corroborated experimentally by recent studies using a bacterial bioreporter for reproductive success [7]. Specifically, it was demonstrated that bacterial immigrants to the leaf surface vary in their ability to produce offspring, suggesting that indeed the leaf consists of sites differing in conduciveness to cluster formation [8].

A major contributing factor to the lateral variation in bacterial clustering on leaf surfaces is the heterogeneous distribution of free water [9]. Without water, bacteria cannot grow, are subject to desiccation stress, and will eventually die [10]. Veins and trichomes retain water longer than other parts of the leaf cuticle [11] and represent sites where bacteria may be better protected from water stress. Also, the prolonged presence of water at these sites may increase the local availability of nutrients. Most leaf nutrients such as sugar photosynthates originate from the plant’s interior and by diffusion through the cuticle end up on the leaf surface [12], [13], where they are used by bacteria on the leaf surface [14]. Water droplets on a leaf surface are effective sinks for the outward diffusion of these sugars [15]. The rate of diffusion is a function not only of the volume of a water droplet and the rate at which bacteria in the droplet consume the sugars, but also the hydrophobicity of the cuticle (which determines the contact angle of the water droplet and thus the area over which sugars may diffuse) and the thickness or composition of the cuticle (which determines its permeability). All these factors are likely to contribute to the heterogeneity in nutrient availability for bacterial colonizers and to the spatial and temporal variation in bacterial cluster sizes.

As a key step towards a more complete understanding of the complexity of water-dependent processes influencing bacterial cluster formation on leaf surfaces, we have developed PHYLLOSIM (after PHYLLOsphere SIMulation). Using an approach known as pattern-oriented modeling which aims to match observed patterns with model-generated patterns and adjusts the processes or parameters of the model in order to improve the match between observed and predicted patterns [16], we simulated different ‘waterscapes’ to test how each affected the diffusion of sugar to the leaf surface and cluster formation by individual bacteria. Previously, Pérez-Velázquez et al. [17] have modeled the growth of bacterial colonies on leaf surfaces by assuming that each colony grows until it reaches a pre-assigned carrying capacity. Carrying capacities of colonies were assumed to be log-normally distributed, thereby imposing a log-normal size distribution as a basic pattern rather than explaining it. Their colony-based approach was also unable to capture the experimental time courses showing trends over 8 days of observation [4], as colonies in the model reached carrying capacity quickly and further dynamics were then driven by stochastic fluctuations. Our approach is not colony-based but individual-based, spatially explicit and more mechanistic in that we do not assume sites to have a certain carrying capacity but model the leakage of nutrients through the plant cuticle by diffusion. The goal of our model was to identify the processes required to reproduce bacterial clustering patterns that were documented in the previously mentioned study by Monier and Lindow [4]. In their study, greenhouse-grown bean plants were inoculated with the bacterium *Pseudomonas syringae* and incubated for up to 8 days under conditions of 100% relative humidity. At this point, bacterial cells were found in a wide range of cluster sizes, from single cells to over 10^4^ cells per cluster. We present our PHYLLOSIM-based findings as a series of observations based on models with increasing complexity, from a simple water film covering the leaf surface, to water drops of different sizes, and inclusion of a scenario in which bacteria were able to detach from existing clusters and relocate to form new clusters elsewhere on the same leaf, as has been suggested recently [17]. We also present an experimental setup that was specifically designed to study the dynamics of surface detachment/reattachment and that involves interrogation of GFP-based bacterial bioreporters in simulated ‘waterscapes’ on agarose surfaces. Combined with PHYLLOSIM, this setup will not only aid our understanding of bacterial dispersion on plant leaves, but also has application potential to the bacterial colonization of other (non)living surfaces.

## Results

**Figure 1:**
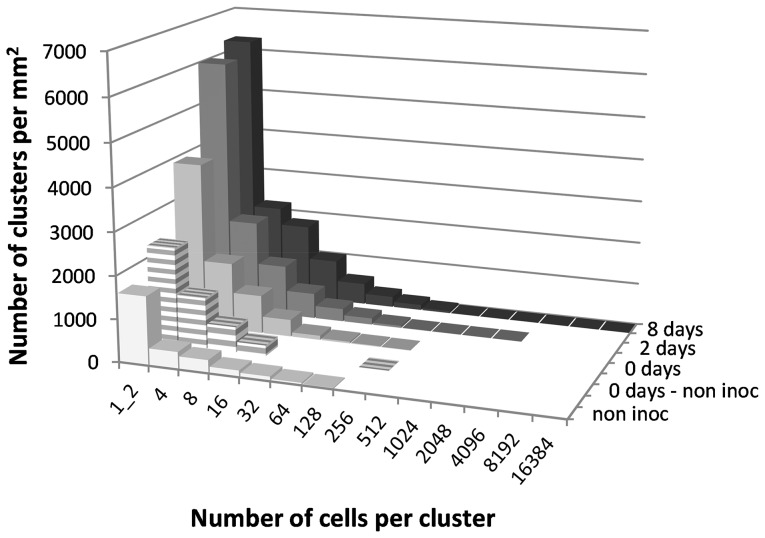
Bacterial cluster sizes on bean leaves, as reported by Monier and Lindow [**4**]. Shown is the frequency distribution of bacterial cluster sizes on leaf surfaces before inoculation (‘non inoc’) and 0, 2, and 8 days after inoculation with the bacterium *P. syringae* pv. *syringae* strain B728a. Data for each time point are an aggregate of measurements from three leaves and were normalized per square millimeter. Also shown is the distribution of cluster sizes of inoculated *P. syringae* cells (‘0 days – non inoc’) which was calculated by subtracting ‘non inoc’ cluster sizes from cluster sizes observed after inoculation with *P. syringae* (‘0 days’). Data shown in this figure are available in Excel format as Supplementary Information (Table S5).

**Figure 2:**
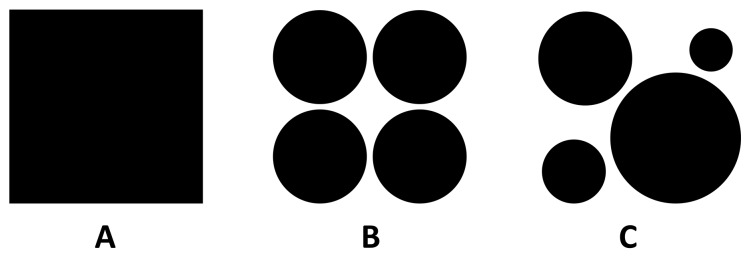
Different ‘waterscapes’ on 1 **mm^2^ of a virtual leaf surface.** Null model: landscape covered by a water film (A), ‘patchy water model’: landscape covered by water drops of the same volume (B) or covered by water drops of different volumes (C). For drop volumes and area coverage, see Table 2.

PHYLLOSIM is an individual-based model of phyllosphere colonization built in part on the previously published model of sugar diffusion across plant leaf cuticles [15]. In PHYLLOSIM, this diffused sugar is consumed by individual bacterial cells, leading to an increase in cell biomass and the production of daughter cells by binary fission. Details of the model, which requires the freely available NetLogo environment to run [18], are described in Table 1 and in the section Materials and Methods, in compliance with the ODD (Overview, Design concepts and Details) framework of Grimm et al. [19]. Our goal was to use PHYLLOSIM to reproduce the experimentally observed bacterial clustering patterns on leaves as reported by Monier and Lindow [4] (Figure 1). PHYLLOSIM consists of a 2-dimensional grid representing 1 mm^2^ of leaf area, onto which virtual bacterial cells were inoculated within the confines of different waterscapes (Figure 2 and Table 2). In the ‘null’ model the water covered the entire leaf surface uniformly as a continuous water film (Figure 2a). In the more complex ‘patchy water’ models, the leaf surface was covered by four equally sized water drops (Figure 2b) or by four drops with different volumes and contact areas (Figure 2c).

**Table 1:** Equations and parameters used in PHYLLOSIM.

Parameter	Symbol	Value	Unit	Reference	Notes
System size		1 * 10^6^	µm^2^		
Grid element size		100	µm^2^		
Maximum growth rate	µ_max_	1.11 * 10^−4^	s^−1^	[14]	Doubling time 1.7 h
Substrate affinity constant	K_s_	0.3	g m^−3^	[14]	
Concentration of sugars in apoplast	C_apo_	18	g m^−3^	[45]	
Permeability of the cuticle	P	2.78 * 10^−10^	m s^−1^	[15]	
Fructose requirement per cell doubling	f	3.0 * 10^−13^	g	[14]	
**Initial conditions**					
Average of the number of bacterial cells per 1 mm^2^ domain	N_0_	10			
Average normalized biomass of each bacterial cell	B_0_	1.5			
**Equations**					
*1)* A = π * sin^2^α* (3*V/(π *(2–3*cosα+cos^3^α)))^2/3^			m^2^	[15]	A = contact area of water drop α = contact angle
*2) C_sink(t+_*_Δ*t)*_ * = (*V * C_sink(t)_+Δ*t (F_(t)_ – U_(t)_))/* V			g m^−3^	[15]	V = volume of water drop
*3) F_(t+_*_Δ*t)*_ * = *A * P * (C_apo_ *- C_sink(t+_*_Δ*t)*_ *)*			g s^−1^	[46]	*F* = Flow of sugar from the apoplast to the sink (water drop)
*4) G_i(t+_*_Δ*t)*_ = B_i(t)_ * µ_max_ * C_sink(t+__Δ*t)*_ */(C_sink(t+_*_Δ*t)*_ *+*K_s_ *)*			s^−1^		Monod kinetics G_i_ = growth of biomass of bacterium i B_i_ = normalized biomass of bacterium i (dimensionless) C_sink_ = Concentration of sugars in the water drop
*5) U_(t+_*_Δ*t)*_ * = *f * Σ*(G_i(t+_*_Δ*t)*_ *)*			g s^−1^	[14]	U = uptake of sugars summed over all bacteria in a water drop
*6) B_i(t+_*_Δ*t)*_ * = B_i(t)_+*Δt * G_i(t+__Δ*t)*_					Δ*t* = time step, 60 s
**Rules**					

1 If B_i_ > = 2, the bacterial cell divides. The biomass is split equally between the parent and daughter cell.

**Table 2:** Water landscape scenarios and corresponding parameters.

Scenario description	Parameter	Symbol	Value	Unit	Reference	Notes
1: water film	Volume water film	V	100 * 10^−12^	m^3^	[9]	
(null model)	Contact area water film	A	100 * 10^−8^	m^2^		The water film is covering the whole simulated leaf area (1 mm^2^)
	Concentration of sugars outsidethe cuticle at t = 0	C_sink_	0	g m^−3^		
2: four water drops ofthe same volume	Volume of each water drop	V	25 * 10^−12^	m^3^	[9]	Sum of volumes of drops keeps the total water volume constant
	Contact area of each water drop	A	13.7 * 10^−8^	m^2^	[15]	Total contact area is 0.55 mm^2^
	Contact angle of each water drop	α	83	rad	[36]	
	Concentration of sugars ineach drop at t = 0	C_sink_	0	g m^−3^		
3: four water dropsof different volume	Volume water drop 1	V1	2.5 * 10^−12^	m^3^	[9]	Sum of volumes of drops keeps the total water volume constant
	Volume water drop 2	V2	7.5 * 10^−12^	m^3^	″	
	Volume water drop 3	V3	22.5 * 10^−12^	m^3^	″	
	Volume water drop 4	V4	67.5 * 10^−12^	m^3^	″	
	Contact area of water drop 1	A1	2.94 * 10^−8^	m^2^	[9]	Total contact area is 0.53 mm^2^
	Contact area of water drop 2	A2	6.12 * 10^−8^	m^2^	″	
	Contact area of water drop 3	A3	17.2 * 10^−8^	m^2^	″	
	Contact area of water drop 4	A4	26.5 * 10^−8^	m^2^	″	
	Contact angle of each water drop	α	83	rad	[36]	
	Concentration of sugars ineach drop at t = 0	C_sink_	0	g m^−3^		

**Figure 3:**
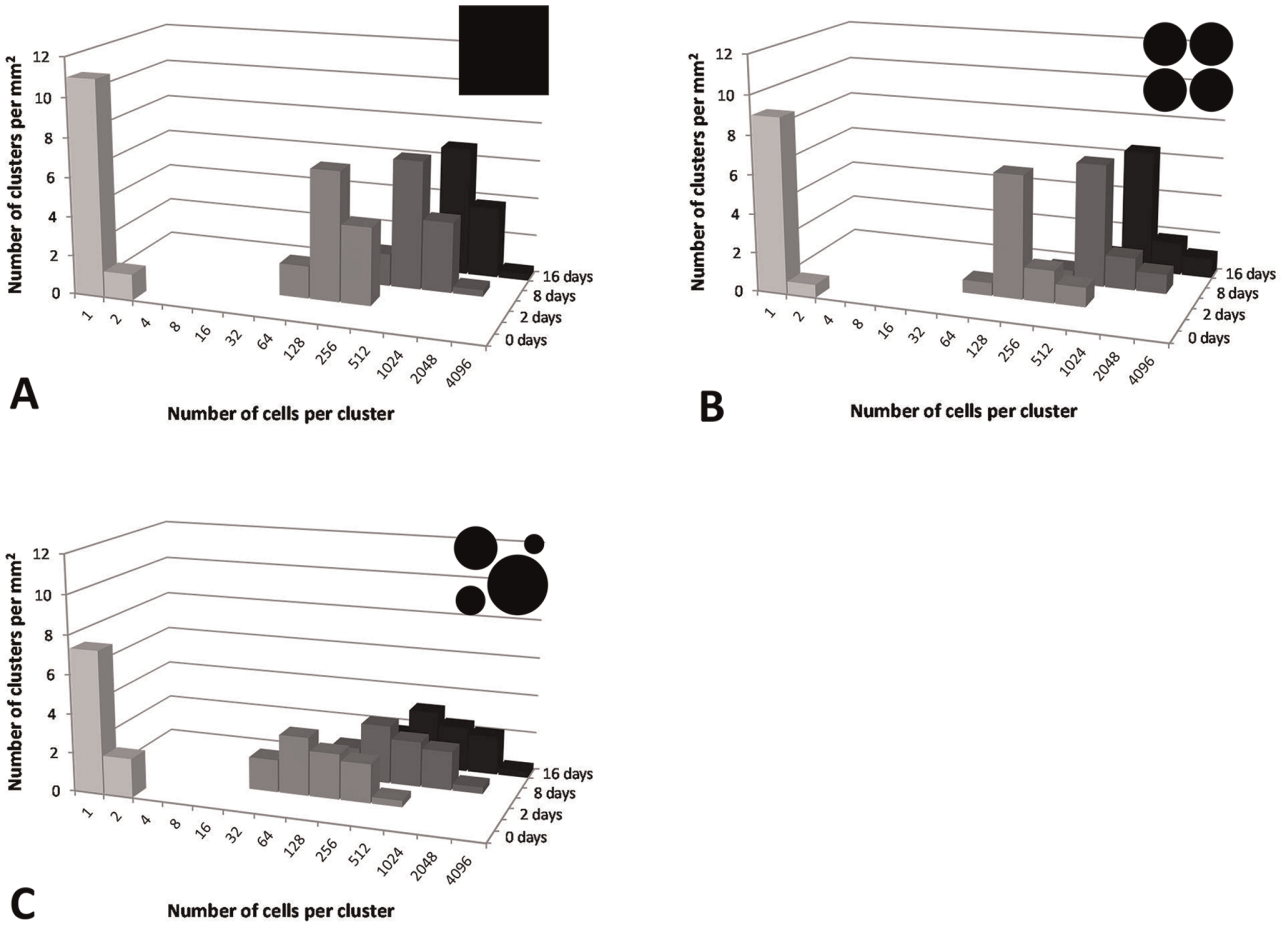
Effect of water distribution on bacterial clustering patterns without detachment, averaged over 3 replicate simulations. A) landscape covered by a water film, B) landscape covered by the same amount of water but in drops of the same volume, C) landscape covered by the same amount of water but in drops of different volumes. Data shown in this figure are available in Excel format (Table S6).

With the PHYLLOSIM ‘null’ model (i.e. continuous water film), bacterial cluster sizes varied between 64 and 256, 256 and 2048, or 512 and 4096 cells after 2, 8, or 16 days of incubation respectively (Figure 3a). In the more complex ‘patchy water’ scenarios, a similar pattern was found (Figure 3b and 3c), although there was more variation in cluster sizes, especially when the leaf was covered with water drops of different volumes. In that case, cluster sizes varied between 32 and 512, 128 and 2048, or 256 and 4096 cells after 2, 8 or 16 days of incubation (Figure 3c). This increase in variation was expected given that different drop volumes result in different contact areas and therefore rates of diffusion across the cuticle and different sugar availabilities to epiphytic bacteria in those drops [15]. Importantly, we note that in all cases, no small clusters existed after day 0. This is a major deviation from the experimental data, which show many single cells or cells in clusters of 2–4 cells throughout the 8 days of observation (Figure 1).

**Figure 4:**
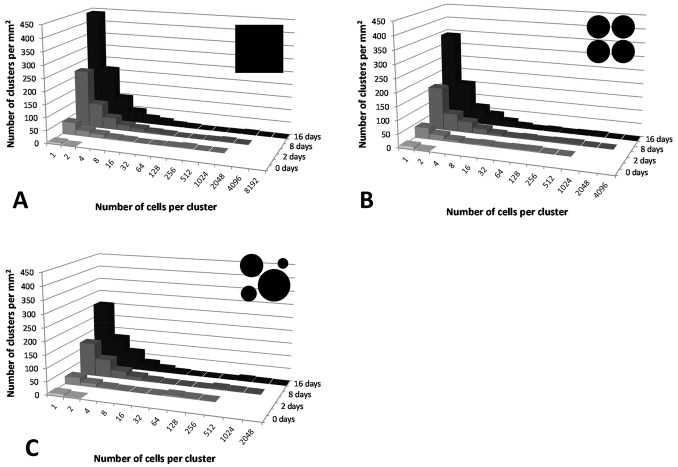
Effect of detachment of cells (probability of 5% after division) on bacterial clustering patterns in combination with effect of water distribution, averaged over 3 replicate simulations. A) landscape covered by a water film, B) landscape covered by water drops of the same volume, C) landscape covered by water drops of different volumes. Data shown in this figure are available in Excel format (Table S1).

**Figure 5:**
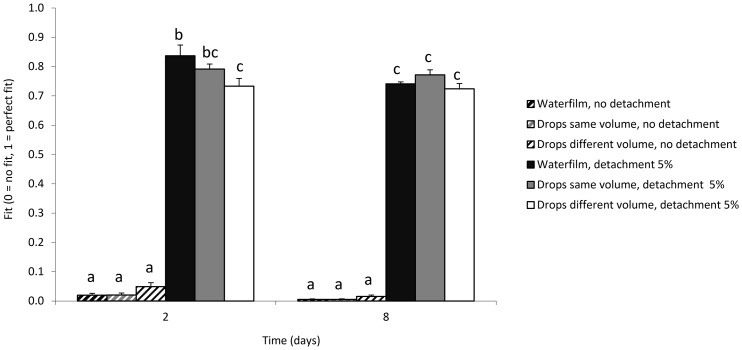
Fit of the frequency distribution of colony size predicted by PHYLLOSIM with those observed by Monier and Lindow [**4**] calculated according to equation (1). Fit (F) ranges from 0 (no fit) to 1 (perfect fit). Different letters indicate significant differences at α = 0.05 among all scenarios. Data shown in this figure are available in Excel format (Table S2).

We repeated the simulations with the three water scenarios but now assuming that single cells could detach from developing clusters. Our hypothesis was that this would result in a better match with the experimental data’s high relative abundance of small clusters. We simulated a range of probabilities of detachment for newly formed cells (2.5%, 5% and 10%), and indeed observed in all simulations an increase in the number of small clusters consisting of only one or two cells (Figure 4). The introduction of detachment also led to an increased range of cluster sizes. For example, with a detachment probability of 5% in the ‘null’ model, cluster sizes varied between 1 and 899, 1 and 3425, or 1 and 6689 cells after 2, 8, or 16 days of incubation, respectively (Figure 4a). A similar effect of detachment was found with the ‘patchy water’ models (Figure 4b and 4c). The patterns were very similar with detachment probabilities of 2.5% and 10% (Table S1 and S2). The main difference was that after 16 days, 2.5% or 10% detachment probabilities resulted in half or twice, respectively, the number of cells in clusters of 1 to 2 cells, compared to 5% detachment. We calculated F, a measure of fit of the simulated versus observed cluster size distributions for each of the six modeled scenarios at 2 and 8 days (Figure 5). Conversely, F values were low for all those scenarios that did not include detachment. Finally, we tested the effect of different initial concentrations of sugars on bacterial cluster sizes, but these did not improve or change patterns of bacterial clustering (Table S3 and S4).

**Figure 6:**
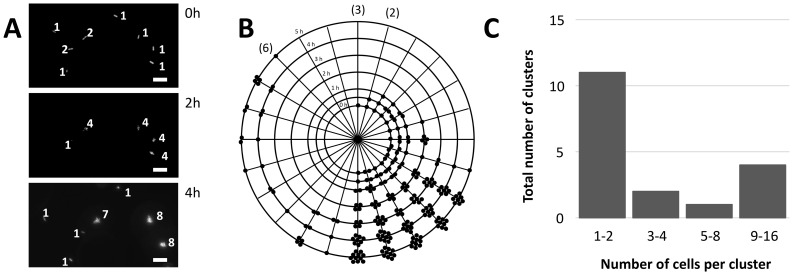
Dynamics of growth, attachment, detachment and re-attachment of *P. agglomerans* 299R::JBA28 at the agarose-water interface. A) Micrographs showing the GFP-tagged bacteria at the surface of the gel medium at different times after inoculation. Cluster sizes are indicated. The bar represents 10 µm. B) Diagram summarizing the cluster dynamics observed in the experiment. Each black dot corresponds to an individual bacterium. Every line represents the fate of an individual immigrant or individual group of immigrants to the agar surface, while concentric circles correspond to different time points after inoculation. Numbers in brackets indicate multiple occurrences of that particular cluster scenario. C) Cluster distribution sizes at t = 5 h. None of the clusters contained more than 16 cells.

**Figure 7:**
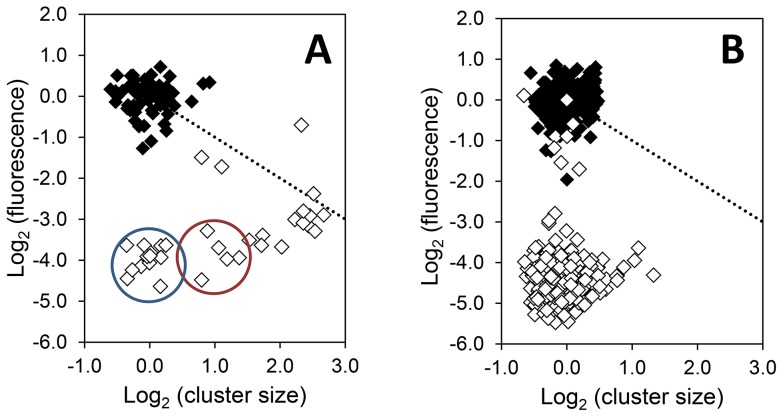
CUSPER-based demonstration of detachment and re-attachment of bacterial cells at the agarose-water interface. Plotted is the mean GFP content of CUSPER cells in a single cluster as a function of the size of that cluster, either as observed on the agarose surface (A) or collected from the liquid in contact with that surface (B). Each diamond represents a cluster for which GFP content and cluster size was recorded at t = 0 h (closed diamonds) or t = 6 h (open diamonds). GFP fluorescence and cluster size were measured by image cytometry and normalized to the average value of the population at t = 0 h. The stippled line represents the expected trend for clusters from which no cells ever detach: with each cell division, the log_2_(fluorescence ) decreases proportionally with the log_2_(cluster size). The blue and red circles in panel A indicate clusters of 1 or 2 cells, respectively, with a GFP content similar to that of the majority of cells in the planktonic phase (panel B, open diamonds).

We designed an experimental setup to allow measurements of surface detachment probabilities. For this, we took advantage of the availability of GFP-based bioreporters derived from the model epiphytic bacterium *Pantoea agglomerans* strain 299R (*Pa*299R). In our setup, cells of *Pa*299R::JBA28 (which constitutively express GFP) were inoculated onto the surface of a flat agarose patch of defined medium containing fructose as a carbon source. This patch was then brought into contact with a droplet of the same medium but devoid of a carbon source. Bacteria could thus occupy either the solid agarose surface or the liquid phase. Bacteria that were attached to the gel medium were visualized over time using epifluorescence microscopy. At the time of inoculation, bacteria were attached to the surface as single cells or as groups of no more than two cells (Figure 6a). When a bacterial cell or group of cells disappeared from the field of view (i.e. the agar surface), we concluded that it had detached from the surface into the liquid phase. During the course of the experiment, several cells remained attached and successfully reproduced, forming clusters of up to 11 cells (Figure 6b). After 3 hours of incubation, single bacteria started to re-attach to the agarose surface, and some of these started to divide (Figure 6b). After 5 hours of incubation, we observed a wide distribution of cluster sizes, with the majority of clusters consisting of 1 or 2 cells (Figure 6c). From the data, using the PHYLLOSIM assumption that only newly formed cells can detach from a cluster, we calculated a detachment probability of approximately 30% for this experimental setup. In a variation on this experiment, we used a derivative of *Pa*299R::JBA28 carrying plasmid pCPP39, which is also known as CUSPER [8] based on its ability to report a cell’s reproductive success through dilution of GFP over consecutive cell divisions. This CUSPER strain features IPTG-inducible expression of GFP, so that cells can be loaded with green fluorescence [7]. Upon release of these cells onto the agarose, no new GFP is formed in the cells (because IPTG is absent) and the previously produced GFP is diluted from cells as a consequence of binary fission. This means that the GFP content of each individual CUSPER cell can be used as a quantitative measure for the number of divisions a cell has undergone. The results of this experiment are presented in Figure 7. Over the course of 6 hours, most clusters contained fewer cells than would be predicted based on the GFP content of cells that make up each cluster, suggesting that these clusters lost cells to the liquid phase. The lack of large clusters in the liquid phase may indicate that single cells rather than larger clusters detach from the surface (Figure 7b). Some cells on the surface occurred in clusters of 1 or 2 cells (Figure 7a, circled in blue or red, respectively) and were similar in GFP content to the majority of cells in the liquid phase, suggesting that they had re-attached recently from the liquid to the surface.

## Discussion

Using PHYLLOSIM, we demonstrated that experimentally observed patterns of bacterial dispersion and cluster sizes on leaf surfaces could not be explained based solely on variation in the patchiness of the leaf waterscape and in the leaching of nutrients which is linked to this variation. While we did not exhaustively test all possible waterscapes, the ones that we did test (i.e. continuous water film, water drops of same size, and water drops of different sizes) resulted in patterns that did not differ much from each other. More importantly, none of the resulting patterns resembled the experimental observation that throughout the initial stages of leaf colonization a large number of cells occur in small clusters. Instead, we found that observed patterns could be recreated by assuming a scenario of bacterial detachment and relocation and that this was more or less independent of the tested waterscape (Figure 4). We note that the experimental data were obtained under conditions of 100% relative humidity, where it is likely that water is retained and covers large areas of the leaf surface as assumed in the model. Under these conditions, detachment and re-attachment of bacteria appear to be sufficient to explain the observed dispersion patterns and variation in cluster sizes.

Pérez-Velázquez et al. [17] recently published a model of phyllosphere colonization. They visually compared model results with observed distributions of bacterial cluster sizes on leaf surfaces. Like our quantitative comparison of model predictions with data, their qualitative comparison clearly indicated the need for detachment to explain the abundance of small clusters. However, they partially imposed the pattern they wanted to explain. Specifically, they assumed that growth of clusters is logistic, where a given cluster can only grow to a maximum size, the carrying capacity, and that these maximum sizes are log-normally distributed. Since growth rate in the model was assumed to be 0.4 h^−1^ the colonies rapidly reached the carrying capacities. Hence, the cluster size distribution in the model changed only due to stochastic processes after the initial 1–2 days, while experimental data show trends over the entire 8 days observed [4]. In contrast, PHYLLOSIM does not make any *a priori* assumptions about the cluster size distribution, such as putting a limit on the number of offspring of a founding cell. Instead, PHYLLOSIM-generated predictions resulted from underlying mechanisms such as attachment, growth by substrate consumption, substrate diffusion, cell division and detachment. In PHYLLOSIM, the ability of founding cells to produce offspring and form clusters depended only on the environment, i.e. the volume of the water drop it landed in and the number of other cells in that drop. Another way in which the model of Pérez-Velázquez et al. [17] differs from PHYLLOSIM is that it is not spatially explicit. This means that it cannot constrain the paths along which nutrients can diffuse or along which detached bacteria may relocate to start a new colony. Such constraints are likely to be in effect on real leaves, for example as enforced by variation in leaf topography. PHYLLOSIM will be much more amenable to the inclusion of such topography in future versions of the model.

Despite their differences, the model of Pérez-Velázquez et al. [17] and PHYLLOSIM independently exposed the role of detachment in phyllosphere colonization by bacteria. From an experimental point of view, the detachment of single cells or small groups of cells from bacterial clusters and their relocation on the same leaf have not been studied extensively. Because detachment and relocation are sudden events that are not easy to quantify *in vivo*, little is known about their relative contribution to bacterial colonization of leaf surfaces. It has been observed that *P. syringae* cells can spread rapidly on wet leaves and are able to colonize areas on the leaf away from the point of inoculation [20]. Laboratory model surfaces that allow regulation of water activity also show the importance of a water film for dispersal [21]. Recently, in a study by Tecon & Leveau [22], use of the CUSPER bioreporter revealed that many leaf surface clusters of the model bacterial epiphyte *P. agglomerans* consisted of fewer cells than would be predicted based on the number of doublings that the cells in a given cluster had undergone. This observation supports the notion that detachment and relocation of bacteria within the waterscape on leaf surfaces are common at least during the early stages of leaf colonization. Our observations of bacterial behavior at the agarose-water interface (Figure 6 and 7a) are consistent with this notion. Although the conditions of our agarose-water experiments differed in important ways from the leaf experiment (i.e. agarose instead of actual leaf surface, *Pantoea agglomerans* instead of *Pseudomonas syringae*), they add to the accumulating evidence that detachment is a process with general importance, not limited to one specific system. For example, detachment is also important in biofilm structure formation and dispersal [23], [24]. Our experiments also demonstrate the utility of our setup as an experimental tool in combination with GFP bioreporter technology to quantify the phenomena of detachment and relocation, which deserve more recognition in experimental designs and conceptual models of how bacteria populate and explore leaf surfaces.

Bacterial detachment from clusters can be a passive or active process. In biofilms, shear stress has been described as a key contributor to cell dispersal [25], [26]. Similar hydrodynamic forces, for example those that occur when water drops evaporate, may also promote bacterial detachment from clusters on the leaf surface. Passive detachment may explain the observations by Hirano et al. [27], who identified a positive impact of raindrop momentum (but not raindrop volume) on the growth of phyllosphere populations of *Pseudomonas syringae* on snap bean under field conditions. Possibly, this momentum caused single cells (or small clumps of cells) to dislodge from established bacterial clusters and to be relocated to other sites on the leaf surface where they form new bacterial clusters. Detachment may also be an active process, representing a response of cells to a changing environment [28], [29]. In biofilms, this response is sometimes linked to nutrient depletion and signal accumulation [30]. On leaf surfaces, limited access of bacteria to sugar [14] and iron [31] has been reported but never explicitly linked to cluster size or to the tendency of cells to leave those clusters. For *P. syringae*, it was shown that the accumulation of quorum sensing molecules such as *N*-acyl homoserine lactones occurs even in small clusters of about 10–40 cells [32] and induces many density-dependent behaviors, but it has not yet been studied in relation to bacterial detachment from clusters. More recently, a transcriptome study of *P. syringae* [33] showed that genes for flagellar motility, swarming motility, chemosensing and chemotaxis are induced during epiphytic growth, indicating definite potential of this bacterium to disperse while on leaf surfaces.

While the ‘detachment’ scenario provided a cluster size distribution that was qualitatively similar to the one found by Monier and Lindow [4], we note that there was a quantitative discrepancy between the model output and empirical data, i.e. Monier and Lindow [4] observed up to 60 times more clusters per size class at 8 days after inoculation. One explanation for this difference could be that our virtual leaves were inoculated with single cells only, whereas the experimental leaves were inoculated with what appeared to be already clusters of various sizes up to 16 cells (see Figure 1, ‘0 days – non inoc’). The inoculum for the experiment was prepared by scraping bacteria from agar plates and resuspending them in water [4]. Incomplete disruption of cell clusters in the suspension thus prepared may explain why many *P. syringae* cells already occurred in clusters at time *t* = 0. Another possible explanation for the model-experiment discrepancy might be that *P. syringae* cells closely packed together in clusters switched to a state that involves the coordinated expression of traits that are regulated by cell density and clustering [34] and that improve access to nutrients. Many phyllosphere bacteria produce surfactants [35], which enhance leaf wetting and decrease the leaf’s contact angle [36], thereby increasing the leaf surface area that is available for nutrient leaching [15]. Bacteria have also been shown to increase cuticle permeability, which further increases diffusion of sugars and other nutrients [37]. Both types of ‘ecosystem engineering’ by leaf surface bacteria could explain an increase in total cells for each of the size categories, but only if the quorum sizes for upregulation of genes were relatively low due to strong clustering or diffusion limitation [34]. Indeed, a quorum size of as low as 13 cells has been reported for *P. syringae* on leaf surfaces [32].

In conclusion, our modeling suggests that dispersion by detachment and re-attachment is the main factor contributing to the observed distribution of clusters sizes on leaves under conditions of high relative humidity. Our experimental system designed to microscopically record attachment and detachment events confirms the existence of such a dispersal process. Under natural conditions, the waterscape is likely to be much more dynamic than we assumed in the model or on the agarose surface, both spatially and temporally. Under such conditions, which may feature events such as evaporation and rain [10], fragmentation and coalescence in the waterscape may result in an even greater degree of variation in bacterial dispersion. In its current form, PHYLLOSIM is not able to deal with such spatiotemporal variation in the waterscape. On the other hand, there are currently few quantitative data sets of bacterial dispersion under dynamic but defined conditions of water availability (e.g. [38]) that could be used to test such a model. Future efforts in this direction should focus on generating such data and on using them to validate and improve PHYLLOSIM for making predictions of bacterial colonization of the phyllosphere, and other unsaturated surfaces, under a wide range of waterscape scenarios.

## Materials and Methods

### Model Description

#### Purpose

The purpose of PHYLLOSIM is to simulate as closely as possible the bacterial clustering patterns in the phyllosphere as reported by Monier and Lindow [4] in order to understand the mechanisms that contribute to the variation in bacterial cluster sizes.

#### Entities, state variables and scales

On a 2D grid representing 1 mm^2^ of leaf area, bacterial cells (the entities) were randomly spread across the leaf surface within the confines of one of several different waterscapes (see below). Edge effects were avoided by applying periodic boundary conditions. In order to keep simulation times reasonable, the 2D grid consisted of 100×100 elements, each representing 100 µm^2^ of leaf. Since we compared our data with empirical data from the study by Monier and Lindow [4], we evaluated output data at the same time points as did these authors, i.e. after 0, 2, and 8 days of incubation, as well as after 16 days of incubation. Time steps of 60 s were used, resulting in 23,040 time steps for each scenario.

In the model, the state of each bacterial cell was characterized by the variables *colony id* and *biomass* (B). A newly formed bacterial cell received the same colony id as its parent cell. If the new cell detached from its original cluster, it would start a new colony with a new colony id so that bacterial cells with the same colony id belong to the same cluster. For each bacterium, biomass B was normalized to fall between 1 and 2 (see below). Each cell’s volume was equal to B * 1 µm^3^, so that with an assumed height of 1 µm [4], each cell occupied an area of B * 1 µm^2^ on a grid element. Clusters were assumed to consist of a monolayer of bacterial cells [4]. If the number of bacterial cells in a grid element exceeded 100, new daughter cells moved randomly to one of the surrounding grid elements. Each body of water (e.g. a water droplet) was characterized by the state variable *concentration of sugars in the water drop* (C_sink_). Since the plants in the experiments of Monier and Lindow [4] were kept at 100% humidity, we assumed that water did not evaporate (i.e. volumes remained the same throughout the experiment).

### Process Overview per Submodel and Scheduling

#### Bacterial processes

We assumed that sugar was the limiting substrate for bacterial growth [14], which was considered to follow Monod kinetics with µ_max_ = 1.11 * 10^−4^ s^−1^ and K_s_ = 0.3 g m^−3^ (Table 1, equation 4). These values were derived for another leaf colonizer, *Pantoea agglomerans* [14], as no such values are available for *P. syringae*. The rates of change of sugar concentration and cells’ biomasses were discretized in time, so a certain concentration of sugar or amount of biomass was consumed or formed per time step, respectively. The consumption of sugars was described by the product of increase in biomass summed over all bacteria separately for each water body and the amount of sugars that a bacterial cell needs to replicate (Table 1, equation 5). Each time step, biomass B was incremented for each individual bacterium (Table 1, equation 6 and rule 1); if it reached or exceeded the value of 2, the bacterial cell divided. Any excess biomass was split equally between the two daughter cells.

#### Diffusion of sugar across the leaf cuticle

We assumed that mass transport (flow) of sugar from the plant’s interior to the leaf cuticle only took place in areas covered by water. The relationship between the contact area, the volume of the water drop, and its contact angle was described by equation 1 (Table 1). Flow per area was proportional to the concentration difference between sugars inside the plant and in the water drops (equation 3, Table 1) and the permeability (*P*) of the leaf cuticle (which was assumed to be uniform across the surface). For *P*, we used values obtained with leaf cuticles of walnut (*Juglans regians*) [15], as no *P* values are available for bean leaf cuticles. We assumed that the rate of change of the concentration of sugar in the water drops was determined by the volume of the water drop, and the rates of flow and uptake of sugar by bacteria (equation 2, Table 1). Since diffusion of small molecules over short distances is fast [39], we assumed a uniform concentration of sugar within each droplet.

### Design Concepts

#### Emergence

The size of bacterial clusters emerges from water-dependent sugar transport and the behavior (growth on sugar and detachment/re-attachment from developing clusters) of individual bacterial cells.

#### Sensing

Bacteria are able to sense the concentration of sugar available for growth.

#### Collectives

Bacteria are grouped into clusters (also referred to in the phyllosphere literature as microcolonies or aggregates).

#### Observation

The biomass of each individual, the colony id, the number of bacteria per water drop, the total number of bacteria and the concentration of sugar per water drop on the leaf over time were recorded. For model analysis, the size of individual bacterial clusters after 0, 2, 8 and 16 days was determined by counting the number of bacteria per cluster.

### Initialization

To inoculate leaves with bacteria, Monier and Lindow [4] immersed leaves in a suspension of 10^5^ bacterial cells/ml. If we assume that 1 mm^2^ of leaf surface was covered by 0.1 µl of water [9], this would result in an average of 10 bacterial cells per mm^2^. Thus, in our simulations, the number of bacterial cells inoculated onto the 1-mm^2^ virtual leaf, was assumed to follow a Poisson distribution [40] with a mean of 10. Since bacteria were inoculated via a suspension, we assumed that all bacterial cells landed on the leaf in water. We also assumed that bacteria and their offspring stayed in the drop in which they arrived. The initial concentration of sugars in the water (C_sink_) was set to 0. To create variation in lag time for cell division of each bacterium (i.e. the time it takes before first division) [41], [42], the initial biomass (B) of bacterial cells was assumed to follow a normal distribution with a mean of 1.5 and a standard deviation of 0.5, but the initial biomass was not allowed to be smaller than 1. Using these values, the time before the first division varied from 0 to 4.5 hours with an average of approximately 2.3 hours. This seemed to be a reasonable average lag time for *P. agglomerans* inoculated on leaves [7]; no data are available for *P. syringae*.

### Simulated Scenarios

Different ‘waterscapes’ (Table 2) on a 1-mm^2^ patch of leaf surface were simulated by keeping the total volume of water (0.1 µl) the same, but by varying the area covered by that water. This rule allowed us to study the impact of different waterscapes independent of total water volume. We started with the simplest assumption, the ‘null’ model, in which the water covered the entire leaf surface uniformly as a continuous water film (Figure 2a). In the more complex ‘patchy water’ models, the leaf surface was covered by four water drops of 0.025 µl each (equally sized drops, Figure 2b) or by four water drops with different volumes and contact areas (Table 2, Figure 2c).

In a factorial design, we also tested these three model scenarios under the additional assumption that bacterial detachment occurred (‘detachment’ model), i.e. after division, a daughter cell had a certain probability of dispersal, to leave the division site and start a new colony by instant re-attachment at a random location within the same water body [21]. Probabilities of detachment of 2.5%, 5% and 10% were tested and random numbers were drawn from a uniform distribution.

### Computational Resources

PHYLLOSIM was written in Netlogo 4.1RC3 [18]. A copy is available on request. All simulations were performed on a HP Compaq Business Desktop dc5800 - Core 2 Duo E8400 3.0 GHz. Three replicate simulations were conducted for each scenario and each run took about 1 computer hour.

### Statistical Analyses

As a measure of fit (F) of the frequency distribution of cluster sizes predicted by PHYLLOSIM to those observed by Monier and Lindow [4], we calculated the summed absolute difference in relative frequency for all cluster size classes (n) according to the equation: 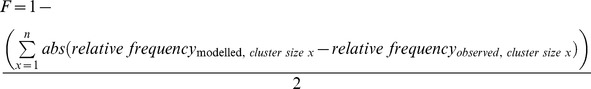



In case of a perfect fit, F equals 1 and in case of a complete mismatch between data and model, F equals 0.

### Experimental Approach

#### Bacterial strain and culture conditions

We chose *Pantoea agglomerans* 299R::JBA28 [43] as a model bacterial strain for testing the detachment hypothesis that emerged from our model simulations. *Pantoea agglomerans* (formerly known as *Erwinia herbicola*) is a common, well-characterized colonizer of leaf surfaces [7]–[9], [14], [15], [22]. The strain was routinely grown at 30°C on Luria Bertani (LB) agar plates or in LB liquid cultures with 50 µg of kanamycin per ml. *Pa*299R::JBA28 is equipped with a chromosomal mini-Tn*5*-Km cassette conferring resistance to kanamycin and containing the *gfp*mut3 gene under the control of the promoter P*_A1/04/03_*, which provides the cells with constitutive expression of a stable green fluorescent protein (GFP). Strain *Pa*299R::JBA28 carrying plasmid pCPP39 is also known as CUSPER [8]. It was maintained with 10 µg of tetracycline per ml to select for the plasmid. The *lacI* ^q^ gene on pCPP39 represses expression of GFP and renders it inducible with IPTG at 1 mM final concentration. In the absence of IPTG, GFP-loaded CUSPER cells dilute GFP at a rate that is proportional to the rate of cell division [7].

#### Bacterial growth and detachment on surfaces

Mid-exponential bacterial cultures of *Pa*299R::JBA28 or CUSPER in LB medium were centrifuged at 2,500 g for 10 min. The cells were washed twice with M9 medium [44] devoid of a carbon source, and resuspended in the same medium to an optical density at 600 nm of approximately 0.02. M9 medium (without carbon source) containing 1% of agarose MP (Roche Diagnostics, Indianapolis, USA) was brought to a boil in a microwave oven until the agarose was fully dissolved, then allowed to cool down to 50°C, followed by addition of fructose and casamino acids to final concentrations of 0.4% and 0.2%, respectively. A 20-µl droplet of this solution was pipetted onto the surface of a 24×50 mm glass coverslip (Fisher Scientific, USA), and covered by a 22×30 mm coverslip (Fisher Scientific, USA) which produces a thin film of agarose between the two cover slips. After a few seconds, the smaller coverslip was removed, leaving the agarose gel as a film on the larger one. Five to ten µl of bacterial suspension (*Pa*299R::JBA28 or CUSPER) were placed by pipet onto the surface of the gel and incubated at room temperature until the liquid had disappeared (approximately 5 min). The coverslip was flipped upside down and put in contact with a 200-µl droplet of M9 (without carbon source) in an incubation chamber (another 24×50 mm coverslip mounted in an aluminum frame and maintained by two cardboard spacers of 25×25×1 mm), which was then sealed with parafilm. Bacteria attached to the surface of the gel were visualized with an Axio Imager M2 microscope (Zeiss, Germany) using a 40× objective (EC Plan-NEOFLUAR 40×/0.75, Zeiss), and the chamber was incubated on the microscope stage at room temperature during the course of the experiment. We focused on single fields of view with dimensions of 222×166 µm. With *Pa*299R::JBA28, we recorded images every hour for 5 hours with an AxioCam MRm monochrome camera (Zeiss), utilizing a GFP filter cube (exciter: 470/40 nm; emitter: 525/50 nm; beamsplitter 495 nm) and an exposure time of 100 ms. For CUSPER cells, we took images of the agar surface at t = 0 h and t = 6 h using phase contrast and the GFP filter cube (200 ms of exposure). We also sampled CUSPER cells in the bacterial inoculum at t = 0 h and in the droplet that was in contact with the gel at t = 6 h, by pipetting 5 µl on a piece of agarose gel, covered it with a coverslip and took images as described above. We analyzed CUSPER images using a macro created in the program Axiovision (version 4.8, Zeiss). Briefly, phase contrast images were used to measure the surface area (in µm^2^) of cell clusters and to create a mask for the analysis of the mean GFP fluorescence intensity in the corresponding cell clusters, expressed in Average Gray Value per unit of exposure time (AGV/ms). Fluorescence intensity and surface area were normalized to the average value in the sampled population at t = 0 h.

## Supporting Information

Table S1 Raw data related to Figure 4.
(XLSX)

Table S2 Raw data related to Figure 5*.
(XLSX)

Table S3 Different initial concentrations of sugars in the water (Csink) were set to test their effect on number of bacteria per size class*.
(XLSX)

Table S4 Fit of the frequency distribution of colony size predicted by PHYLLOSIM with those observed by Monier and Lindow (2004) calculated according to equation (1)*.
(XLSX)

Table S5 Raw data related to Figure 5*.
(XLSX)

Table S6 Raw data related to Figure 3*.
(XLSX)

## References

[1] Lindow SE, Brandl MT (2003) Microbiology of the phyllosphere. Appl Environ Microbiol 69: 1875–1883. 12676659 10.1128/AEM.69.4.1875-1883.2003 PMC154815

[2] Leveau JHJ (2006) Microbial communities in the phyllosphere. In: Riederer M, Muller C (eds) Biology of the plant cuticle, Blackwell Publishing, pages 334–367.

[3] Morris CE, Monier J-M (2003) The ecological significance of biofilm formation by plant-associated bacteria. Annu Rev Phytopathol 41: 429–453. 12730399 10.1146/annurev.phyto.41.022103.134521

[4] Monier J-M, Lindow SE (2004) Frequency, size, and localization of bacterial aggregates on bean leaf surfaces. Appl Environ Microbiol 70: 346–355. 14711662 10.1128/AEM.70.1.346-355.2004 PMC321242

[5] Monier J-M (2006) Bacterial assemblages on plant surfaces. In: Bailey MJ, Lilley AK, Timms-Wilson TM, Spencer-Phillips PTN (eds) Microbial ecology of aerial plant surfaces, CABI (Oxfordshire, UK), pages 83–105.

[6] Monier J-M, Lindow SE (2003) Differential survival of solitary and aggregated bacterial cells promotes aggregate formation on leaf surfaces. Proc Natl Acad Sci USA 100: 15977–15982. 14665692 10.1073/pnas.2436560100 PMC307678

[7] Remus-Emsermann MNP, Leveau JHJ (2010) Linking environmental heterogeneity and reproductive success at single-cell resolution. ISME J 4: 215–222. 19865185 10.1038/ismej.2009.110

[8] Remus-Emsermann MNP, Tecon R, Kowalchuk GA, Leveau JHJ (2012) Variation in local carrying capacity and the individual fate of bacterial colonizers in the phyllosphere. ISME J 6: 756–765. 22258099 10.1038/ismej.2011.209 PMC3309366

[9] Axtell CA, Beattie GA (2002) Construction and characterization of a *proU*-*gfp* transcriptional fusion that measures water availability in a microbial habitat. Appl Environ Microbiol 68: 4604–4612. 12200319 10.1128/AEM.68.9.4604-4612.2002 PMC124082

[10] Beattie GA (2011) Water relations in the interaction of foliar bacterial pathogens with plants. Annu Rev Phytopathol 49: 533–555. 21438680 10.1146/annurev-phyto-073009-114436

[11] Brewer CA, Smith WK, Vogelmann TC (1991) Functional interaction between leaf trichomes, leaf wettability and the optical properties of water droplets. Plant Cell Environ 14: 955–962.

[12] Tukey HB (1966) Leaching of metabolites from aboveground plant parts and its implications. Bull Torrey Bot Club 93: 385–401.

[13] Schreiber L (2005) Polar paths of diffusion across plant cuticles: new evidence for an old hypothesis. Ann Bot 95: 1069–1073. 15797897 10.1093/aob/mci122 PMC4246894

[14] Leveau JHJ, Lindow SE (2001) Appetite of an epiphyte: Quantitative monitoring of bacterial sugar consumption in the phyllosphere. Proc Natl Acad Sci USA 98: 3446–3453. 11248098 10.1073/pnas.061629598 PMC30673

[15] Van der Wal A, Leveau JHJ (2011) Modeling sugar diffusion across plant leaf cuticles: the effect of free water on substrate availability to phyllosphere bacteria. Environ Microbiol 13: 792–797. 21091864 10.1111/j.1462-2920.2010.02382.x

[16] Grimm V, Revilla E, Berger U, Jeltsch F, Mooij WM, et al (2005) Pattern-oriented modeling of agent-based complex systems: lessons from ecology. Science 310: 987–991. 16284171 10.1126/science.1116681

[17] Pérez-Velázquez J, Schlicht R, Dulla G, Hense BA, Kuttler C, et al (2012) Stochastic modeling of *Pseudomonas syringae* growth in the phyllosphere. Math Biosci 239: 106–116. 22659411 10.1016/j.mbs.2012.04.009

[18] Wilensky U (1999) NetLogo. http://ccl.northwestern.edu/netlogo/. Center for Connected Learning and Computer-Based Modeling, Northwestern University, Evanston, IL.

[19] Grimm V, Berger U, Bastiansen F, Eliassen S, Ginot V, et al (2006) A standard protocol for describing individual-based and agent-based models. Ecol Model 198: 115–126.

[20] Leben C, Schroth MN, Hildebrand DC (1970) Colonization and movement of *Pseudomonas syringae* on healthy bean seedlings. Phytopathology 60: 677–680.

[21] Dechesne A, Wang G, Gülez G, Or D, Smets BF (2010) Hydration-controlled bacterial motility and dispersal on surfaces. Proc Natl Acad Sci USA 107: 14369–14372. 20660312 10.1073/pnas.1008392107 PMC2922541

[22] Tecon R, Leveau JHJ (2012) The mechanics of bacterial cluster formation on plant leaf surfaces as revealed by bioreporter technology. Environ Microbiol 14: 1325–1332. 22364368 10.1111/j.1462-2920.2012.02715.x

[23] Xavier JB, Picioreanu C, Van Loosdrecht MCM (2005) A general description of detachment for multidimensional modeling of biofilms. Biotechnol Bioeng 91: 651–669. 15918167 10.1002/bit.20544

[24] Picioreanu C, Kreft J-U, Klausen M, Haagensen JAJ, Tolker-Nielsen T, et al (2007) Microbial motility involvement in biofilm structure formation–a 3D modeling study. Water Sci Technol 55: 337–343. 17547003 10.2166/wst.2007.275

[25] Stoodley P, Lewandowski Z, Boyle JD, Lappin-Scott HM (1999) Structural deformation of bacterial biofilms caused by short-term fluctuations in fluid shear: an in situ investigation of biofilm rheology. Biotechnol Bioeng 65: 83–92. 10440674

[26] Picioreanu C, van Loosdrecht MC, Heijnen JJ (2001) Two-dimensional model of biofilm detachment caused by internal stress from liquid flow. Biotechnol Bioeng 72: 205–18. 11114658

[27] Hirano SS, Baker LS, Upper CD (1996) Raindrop momentum triggers growth of leaf-associated populations of *Pseudomonas syringae* on field-grown snap bean plants. Appl Environ Microbiol 62: 2560–2566. 16535362 10.1128/aem.62.7.2560-2566.1996 PMC1388900

[28] Rice SA, Koh KS, Queck SY, Labbate M, Lam KW, et al (2005) Biofilm formation and sloughing in *Serratia marcescens* are controlled by quorum sensing and nutrient cues. J Bacteriol 187: 3477–3485. 15866935 10.1128/JB.187.10.3477-3485.2005 PMC1111991

[29] Thormann KM, Saville RM, Shukla S, Spormann AM (2005) Induction of rapid detachment in *Shewanella oneidensis* MR-1 biofilms. J Bacteriol 187: 1014–1021. 15659679 10.1128/JB.187.3.1014-1021.2005 PMC545703

[30] Shrout JD, Chopp DL, Just CL, Hentzer M, Givskov M, et al (2006) The impact of quorum sensing and swarming motility on *Pseudomonas aeruginosa* biofilm formation is nutritionally conditional. Mol Microbiol 62: 1264–1277. 17059568 10.1111/j.1365-2958.2006.05421.x

[31] Joiner DC, Lindow SE (2000) Heterogeneity of iron bioavailability on plants assessed with a whole-cell GFP-based bacterial biosensor. Microbiology 146: 2435–2445. 11021920 10.1099/00221287-146-10-2435

[32] Dulla G, Lindow SE (2008) Quorum size of *Pseudomonas syringae* is small and dictated by water availability on the leaf surface. Proc Natl Acad Sci USA 105: 3082–3087. 18287070 10.1073/pnas.0711723105 PMC2268588

[33] Yu X, Lund SP, Scott RA, Greenwald JW, Records AH, et al (2013) Transcriptional responses of *Pseudomonas syringae* to growth in epiphytic versus apoplastic leaf sites. Proc Natl Acad Sci USA 110: E425–E434. 23319638 10.1073/pnas.1221892110 PMC3562829

[34] Hense BA, Kuttler C, Müller J, Rothballer M, Hartmann A, et al (2007) Does efficiency sensing unify quorum and diffusion sensing? Nat Rev Microbiol 5: 230–239. 17304251 10.1038/nrmicro1600

[35] Bunster L, Fokkema HJ, Schippers B (1989) Effect of surface activity of *Pseudomonas* spp. on leaf wettability. Appl Environ Microbiol 55: 1340–1345. 16347926 10.1128/aem.55.6.1340-1345.1989 PMC202868

[36] Knoll D, Schreiber L (2000) Plant-microbe interactions: wetting of ivy (*Hedera helix L.*) leaf surfaces in relation to colonization by epiphytic microorganisms. Microb Ecol 40: 33–42. 10977875 10.1007/s002480000012

[37] Schreiber L, Krimm U, Knoll D, Sayed M, Auling G, et al (2005) Plant–microbe interactions: identification of epiphytic bacteria and their ability to alter leaf surface permeability. New Phytol 166: 589–594. 15819920 10.1111/j.1469-8137.2005.01343.x

[38] Wang G, Or D (2013) Hydration dynamics promote bacterial coexistence on rough surfaces. ISME J 7: 395–404. 23051694 10.1038/ismej.2012.115 PMC3554404

[39] Stein WD (1990) Channels, carriers and pumps: an introduction to membrane transport. Academic Press.

[40] Benedek GB, Villars FMH (2000) Physics with illustrative examples from medicine and biology. Springer Verlag, New York.

[41] Dens EJ, Bernaerts K, Standaert AR, Kreft JU, Van Impe JF (2005) Cell division theory and individual-based modeling of microbial lag. Part II. Modeling lag phenomena induced by temperature shifts. International Journal of Food Microbiology 101: 319–332. 15913823 10.1016/j.ijfoodmicro.2004.11.017

[42] Elfwing A, LeMarc Y, Baranyi J, Ballagi A (2004) Observing growth and division of large numbers of individual bacteria by image analysis. Appl Environ Microbiol 70: 675–678. 14766541 10.1128/AEM.70.2.675-678.2004 PMC348858

[43] Leveau JHJ, Lindow SE (2001) Predictive and interpretive simulation of green fluorescent protein expression in reporter bacteria. J Bacteriol 183: 6752–6762. 11698362 10.1128/JB.183.23.6752-6762.2001 PMC95514

[44] Sambrook J, Russell DW (2001) Molecular Cloning: A Laboratory Manual. Cold Spring Harbor Laboratory Press: New York.

[45] Lohaus G, Winter H, Riens B, Heldt HW (1995) Further-studies of the phloem loading process in leaves of barley and spinach – the comparison of metabolite concentrations in the apoplastic compartment with those in the cytosolic compartment and in the sieve tubes. Bot Acta 108: 270–275.

[46] Schönherr J, Baur P (1996) Cuticle permeability studies - A model for estimating leaching of plant metabolites to leaf surfaces. Plenum Press Div Plenum Publishing Corp: New York.

